# Evaluation of efficacy and safety of combined rosuvastatin and atorvastatin in treating with coronary heart disease: A protocol for systematic review and meta-analysis

**DOI:** 10.1097/MD.0000000000026340

**Published:** 2021-06-18

**Authors:** Ke Li, Meng-Meng Liu, Xin Yang, Li Chen, Hui Geng, Wei Luo, Jie Ma

**Affiliations:** a Department of Hematology, the Affiliated Hospital of Qinghai University, Xining, Qinghai; b Department of Cardiology, the Wanyuan Central Hospital, Wanyuan, Sichuan, China.

**Keywords:** atorvastatin, coronary heart disease, efficacy, rosuvastatin

## Abstract

**Background:**

Globally, coronary heart disease (CHD) is a primary cause of morbidity leading to disabilities and mortality. Modern clinical practice adopts several pharmacological methods to treat CHD. Angina pectoris refers to sever chest pain due to CHD, it has a profound impact on the wellbeing of patients. Moreover, angina pectoris is a crucial prognosis predictor. The aim of the current study is to evaluate the effectiveness and safeness of using combined rosuvastatin and atorvastatin to treat CHD patients.

**Methods:**

A systematic literature search for articles will be conducted on several electronic databases from their inception to May 2021. The search will include all randomized controlled trials examining the use of rosuvastatin in combination with atorvastatin to treat CHD patients. The databases are as follows: MEDLINE, Web of Science, the Cochrane Library, WanFang database, China National Knowledge Infrastructure, and EMBASE. A couple of authors will independently assess the eligibility, extract study data, and assess the possibility of bias. Moreover, depending on the type of data and heterogeneity of the included studies, either the Mantel–Haensel fixed-effect model or the DerSimonian-Laird random-effect model will be used to estimate the relative risk, mean differences, or standardized mean differences and 95% confidence intervals. All differences in opinion shall be decided by involving an additional author in the discussion. Lastly, the RevMan software (version: 5.3) will be used to perform sensitivity analysis, data synthesis, and risk of bias assessment.

**Results:**

The effectiveness and security of using rosuvastatin in combination with atorvastatin to treat CHD patients will be systematically evaluated.

**Conclusion:**

This study will provide evidence to evaluate the efficacy and security of using a combination of rosuvastatin and atorvastatin to treat CHD patients.

**Ethics and dissemination:**

Ethical approval will not be required since it is based on already published data.

**Registration number:**

DOI 10.17605/OSF.IO/VYBDR (https://osf.io/vybdr/).

## 1 Introduction

Coronary heart disease (CHD) is a clinical term used to identify coronary arterial stenosis, it is more commonly known as coronary heart disease. Generally, progressive coronary atherosclerotic lesions block arteries in the heart, causing necrosis or myocardial ischemia.^[1,2]^ CHD is identified as the primary cause of worldwide cardiovascular conditions.^[3]^ Considering the high prevalence and fatality rate associated with CHD, it poses a significant threat to public health.^[4]^ The continuous retaining and accumulation of particles containing cholesterol-abundant apolipoprotein B in the arterial intima triggers atherogenesis.^[5,6]^ Reportedly, atherosclerosis is identified as an inflammatory condition, and prevailing illnesses (i.e., high blood pressure and abnormal lipoprotein content) elevate the risk of incidence and advancement of inflammation.^[7]^ The American Heart Association reported that in 2020, those with CHD face an elevated risk of facing recurrent coronary conditions.^[8]^ Dyslipidemia, hypertension, obesity, overweight, and hypercholesterolemia are the primary reasons behind recurring coronary conditions.^[9]^

Past researches have indicated vast improvements in the treatment and subsequent prognosis of cardiovascular conditions, particularly under strict regulation of Low-Density Lipoprotein Cholesterol (LDL-C).^[10–12]^ The literature associated with the topic is vast and includes studies spanning many decades, which includes prospective epidemiological studies, genetic studies, mendelian randomization studies, and randomized controlled trials, these have conclusively demonstrated that LDL is a casual influencing factor for coronary conditions.^[13]^ Despite the increasing number of patients getting statins, the targeted rate of LDL-C achievement remains considerably low among both Western and Asian populations.^[14–16]^ Many results have demonstrated that in comparison with atorvastatin, rosuvastatin has a stronger effect on reducing blood fat.^[17,18]^ Applying rosuvastatin during the initial phase effectively regulates blood lipids, reverses atherosclerotic plaque, and reduces the atherosclerosis inflammatory index. Collectively, these also lessen the risk of cardiovascular events following percutaneous coronary intervention. Furthermore, there was lesser incidence of adversities compared to other identical medication. Recently, there has been an increasing number of clinical studies that have examined the use of rosuvastatin in combination with atorvastatin to treat CHD. However, it is still not clear whether this treatment plan can effectively improve the symptoms of patients, and whether its safety is reliable. Therefore, we will plan to conduct the present meta-analysis to assess the efficacy and security of using rosuvastatin combined with atorvastatin to treat CHD patients.

## 2 Methods

The current meta-analysis is registered on the Open Science Framework (OSF) (https://osf.io) with DOI 10.17605/OSF.IO/VYBDR. The study is conducted according to the Preferred Reporting Items for Systematic Reviews and Meta-Analyses Protocols (PRISMA-P) statement.

### 2.1 Eligibility criteria of inclusion of studies

#### 2.1.1 Types of studies

We will include all the randomized controlled trials studying the combination of rosuvastatin and atorvastatin for treating CHD.

#### 2.1.2 Types of participants

The participants include patients of all ages and both sexes who have been clinically diagnosed with CHD.

#### 2.1.3 Types of interventions

Randomized controlled trials involving single agent therapy (rosuvastatin alone, atorvastatin alone, placebo, or no treatment) or combination therapy (rosuvastatin in combination with atorvastatin).

#### 2.1.4 Types of outcome measures

The primary outcomes include electrocardiogram changes, severity of angina pectoris, dose of nitro-glycerine, and frequency of acute angina. The minor outcomes include total cholesterol, LDLC, high-density lipoprotein cholesterol levels, triglyceride, and adverse events.

### 2.2 Search methods for study inclusion

#### 2.2.1 Electronic searches

A systematic literature search for articles will be conducted on several electronic databases from their inception to May 2021. The databases are as follows: MEDLINE, Web of Science, the Cochrane Library, WanFang database, China National Knowledge Infrastructure, and EMBASE. The search will identify all randomized controlled trials evaluating the effectiveness and safeness of using rosuvastatin in combination with atorvastatin to treat CHD. The following search terms are used: rosuvastatin∗, atorvastatin∗, “coronary heart disease,” and “randomized controlled trials.”

#### 2.2.2 Searching other resources

An additional search of the reference lists of selected studies will help identify other potentially related studies.

### 2.3 Data collection and analysis

#### 2.3.1 Selection of studies

**Figure 1:**
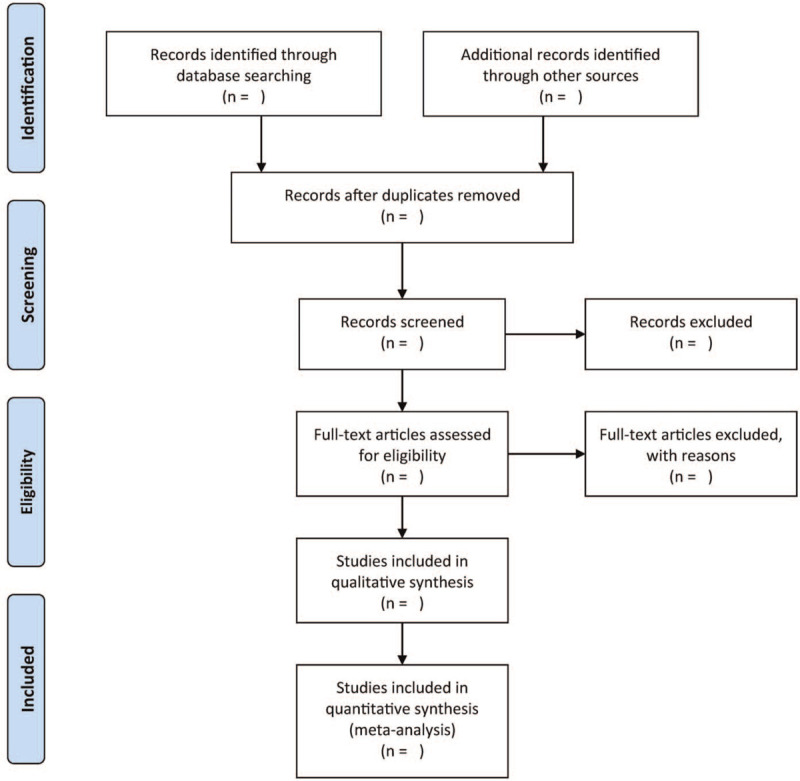
The research flowchart.

A couple of independent authors will screen the titles/ abstracts in the preliminary review. Besides, a pair of autonomous authors will scrutinize the titles/abstracts of the identified studies in the updated review. All disagreements shall be mediated via discussion that includes the opinion of a third author. The flowchart will be demonstrated in Figure 1.

#### 2.3.2 Data extraction and management

In the preliminary review, 2 authors will adopt a standardized, agreed data extraction form to independently extract and collect study data from the chosen studies. The process will be replicated by 3 authors in the updated review. All disagreements shall be mediated via discussion that includes the opinion of a third author.

#### 2.3.3 Assessment of risk bias

Two authors will independently use the Cochrane Collaborations’ tool to assess the systematic quality of the selected trials. We will rate the risk of selection bias by evaluating allocation concealment and randomization. All disagreements shall be mediated via discussion that includes the opinion of a third author.

#### 2.3.4 Measures of treatment effect

For dichotomous outcomes, this study will calculate the relative risk and 95% confidence intervals. For continuous outcomes, we will plan to calculate mean differences or standardized mean differences and 95% CI.

#### 2.3.5 Assessment of heterogeneity

The authors will adopt Chi^2^ and *I*^2^ statistics to explore statistical heterogeneity. We set an *I*^2^ greater than 50% and *P* less than .1 as high heterogeneity, in which case the DerSimonian-Laird random-effect model will be used for analysis; otherwise, the Mantel–Haensel fixed-effect model will be used.

#### 2.3.6 Assessment of reporting biases

The authors will employ a funnel plot to evaluate the reporting bias of the selected studies. Furthermore, we will also use Egger tests to evaluate the funnel plot symmetry.

#### 2.3.7 Assessment of reporting biases

The authors also plan to perform a sensitivity analysis to evaluate the robustness of our findings if applicable.

## 3 Discussion

Recently, there has been an increase in the attention paid to researches on effects of statins in treating patients with CHD. Admittedly, the beneficial outcomes of statins in the treatment of CHD patients have been widely reported. However, the effectiveness and safeness of rosuvastatin in combination with atorvastatin to treat CHD has not been systematically evaluated, which remains a hot issue in terms of clinical practice. Therefore, it is imperative to conduct this study to investigate the efficacy and safety of using a combination of rosuvastatin and atorvastatin to treat CHD patients. Our findings will inform our understanding and provide helpful evidence for clinical practitioners and as a reference for future studies.

## Author contributions

**Conceptualization:** Jie Ma.

**Data curation:** Ke Li, Li Chen.

**Formal analysis:** Ke Li, Meng-Meng Liu, Xin Yang, Wei Luo.

**Funding acquisition:** Meng-Meng Liu, Xin Yang, Hui Geng, Wei Luo, Jie Ma.

**Investigation:** Ke Li.

**Methodology:** Meng-Meng Liu, Xin Yang, Li Chen, Hui Geng, Jie Ma.

**Project administration:** Xin Yang, Hui Geng, Wei Luo.

**Resources:** Li Chen.

**Software:** Ke Li, Meng-Meng Liu.

**Validation:** Ke Li, Meng-Meng Liu, Xin Yang.

**Visualization:** Hui Geng, Wei Luo, Jie Ma.

**Writing – original draft:** Ke Li.

**Writing – review & editing:** Jie Ma.
